# The relative age effect and transition rates across a national soccer program in male and female youth to senior players. A longitudinal analysis

**DOI:** 10.5114/biolsport.2026.154941

**Published:** 2025-10-01

**Authors:** Daniel Nisbet, Mauro Mandorino, Piotr Zmijewski, Toni Modric, José Eduardo Teixeira, Alexandre Moreira, Ryland Morgans

**Affiliations:** 1 School of Sport and Health Sciences, Cardiff Metropolitan University, Cardiff, UK; 2 Al Ahli Saudi FC, Jeddah, Saudi Arabia; 3 Performance and Analytics Department, Parma Calcio 1913, 43121 Parma, Italy; 4 Department of Movement, Human and Health Sciences, University of Rome “Foro Italico”, 00135 Rome, Italy; 5 Jozef Pilsudski University of Physical Education in Warsaw, 00-809 Warsaw, Poland; 6 Research and Development Center Legia Lab, Legia Warszawa, Poland; 7 Faculty of Kinesiology, University of Split, Split, Croatia; 8 High Performance Sport Center, Croatian Olympic Committee, Zagreb, Croatia; 9 Department of Sports Sciences, Polytechnic of Guarda, 6300-559 Guarda, Portugal; 10 Department of Sports Sciences, Polytechnic of Cávado and Ave., 4800-058 Guimarães, Portugal; 11 SPRINT—Sport Physical Activity and Health Research & Innovation Center, 6300-559 Guarda, Portugal; 12 Department of Sport, School of Physical Education and Sport, University of São Paulo, São Paulo, Brazil

**Keywords:** Youth players, Senior players, Soccer, International teams, Relative age effect

## Abstract

This study aimed to: (1) investigate the prevalence and magnitude of the Relative Age Effect (RAE) across a national team program; (2) assess the transition rates from youth to senior international level, and (3) examine the influence of birth quartile distribution on transition. 1518 male and 487 female soccer players from a national soccer association were examined. All participants were divided into birth quartiles: January– March (BQ1), April–June (BQ2), July–September (BQ3), and October–December (BQ4). Significant RAE were observed across all age groups, with small-to-medium effect sizes (Cramer’s V: 0.10–0.19), except for male U-16, female U-16, and U-19 players. Senior teams exhibited the most significant RAE, with odds ratios of 2.27 for male and 1.50 for female players. Transition rates from youth to senior teams were higher for female (30%) than male (20%) players. Contrasting trends by birth quartile were evident with males having the lowest transition rate in BQ1 (17%), while females had the lowest transition rate in BQ4 (25%). The number of youth team appearances in both sexes was a significant predictor of transition probability (β = 0.177), with each additional appearance increasing the likelihood of progressing to the senior team by 19.4%. A significant interaction between sex and youth team appearances indicated that the impact of playing opportunities was less pronounced for male players than females. These findings highlight the influence of the RAE in talent development and underscore the need for equitable opportunities for all players, regardless of sex.

## INTRODUCTION

In youth sports, the Relative Age Effect (RAE) is a well-documented phenomenon whereby athletes born earlier in the selection year are more likely to be selected and succeed due to advantages in physical, cognitive, and psychological development [1–2]. This effect is particularly evident in male athletes aged 15 to 18 years, particularly those participating in soccer and basketball [3–4]. The selection year in most sporting environments typically commences on 1^st^ January and is utilized to categorize players into age groups for training and competition [5]. Additionally, in elite national team soccer, the competitive calendar spans from January to January, which may also influence the RAE.

Athletes born closer to the cut-off date often demonstrate greater maturity in physical, cognitive, and psychological domains compared to younger peers [6–7]. This maturity often provides older athletes with a competitive edge, leading to over-representation in youth teams [4, 8]. Conversely, younger athletes may experience lower performance levels and higher dropout rates prior to realizing full potential [9].

Recent research has identified the RAE in younger athletes aged 6 to 12 years, particularly in sports such as hockey, soccer, and alpine skiing [4, 10]. In these early stages, older athletes often benefit from advanced biological maturity, which enhances physical and cognitive abilities [5]. The RAE has been found most prevalent in male athletes aged 15 to 18 years, especially in elite Swedish male and female youth soccer players [11] and notable discrepancies in birth quartile distributions between male and female national soccer squads [4]. However, research examining the RAE impact on male and female international soccer players and the transition rate from youth to senior international level, and whether birth quartile distribution influences this transition remains limited, though emerging studies are attempting to address this gap [3–4].

For adolescent athletes, the RAE can partially be explained by the earlier onset of puberty in older peers, which provides advantages in strength, speed, endurance, and bone density due to increased testosterone levels [12]. Although this physiological advantage is more pronounced in males [13], it also applies to females, albeit with different hormonal influences and developmental timelines. Thus, the RAE may affect female athletes differently, as variations in physical maturity during adolescence can impact selection and performance in sports [14]. Interestingly, late-born athletes that mature early may still succeed in highly competitive environments [15–16]. As the pool of late-maturing individuals diminishes with age, those who develop later may be under-represented at higher levels of competition [8]. Therefore, the impact of the RAE in both male and female athletes, considering the distinct developmental trajectories of each group warrants further research.

In soccer, the RAE is particularly prevalent, with an over-representation of athletes born in the first quarter of the year (January– March) and under-representation of those born in the final quarter (October–December) [17–18]. Williams et al. [19] found that 40% of players competing in the FIFA U-17 World Cup (1997–2007) were born in the first quarter of the year (January–March), compared to only 16% in the last quarter (October–December). Similarly, Ibáñez et al. [17] identified a significant RAE in five European male soccer leagues, with twice as many players born in January compared to December. This suggests that late-born players face systemic barriers, further reducing the chance of re-integration into soccer following the exclusion from youth programs due to delayed maturity.

Recent studies have confirmed the persistence of the RAE in elite German [37] and Scottish [20] male soccer players. However, in some European environments, the RAE appears less pronounced in older players compared to younger ones [5]. In Italian national youth soccer, only 17% of players selected for youth teams progressed to the senior national team, highlighting a significant turnover and the presence of the RAE in U-17 and U-19 categories [21]. Notably, no differences in transition rates were observed among birth quartiles in Italian national female soccer players, suggesting that the RAE is sex-specific [21].

Despite extensive research into the RAE in various sporting contexts, there is limited research examining its prevalence and impact on transition rates from youth to senior level in elite male and female national soccer players [22]. Thus, this study aimed to: (1) investigate the prevalence and magnitude of the Relative Age Effect (RAE) across a national team program; (2) assess the transition rates from youth to senior international level, and (3) examine the influence of birth quartile distribution on transition. The study hypothesis was that the RAE will be evident in national team soccer, with an over-representation of players born in the first quartile (January–March) and an under-representation of those born in the last quartile (October–December) [17–19]. Additionally, it is expected that players born earlier in the selection year will have a greater likelihood of transitioning from youth teams (U-16 to U-19) to senior international levels compared to those born later in the calendar year [3–4]. The impact of RAE on transition rates is anticipated to be more pronounced in male soccer players than in female players due to differences in biological maturity and selection processes [11, 12, 22].

## MATERIALS AND METHODS

### Participants

One thousand five hundred and eighteen male and four hundred and eighty seven female soccer players from the Football Association of Wales were assessed. The sample players were based on national team selection at specific age groups during 1883–2024. The study cohort consisted of male national team players classified into age groups: U-16 (n = 20), U-17 (n = 522), U-19 (n = 474), U-21 (n = 489), and senior (n = 761). Female national team players were also classified into the following age groups: U-16 (n = 21), U-17 (n = 244), U-19 (n = 271), and senior (n = 205). The number of participants in each age group varied from 20 to 751 and was determined by players that competed in national team fixtures between 1883 and 2024.

All participants were further divided into four quartiles according to the month of birth: January to March, first quartile (BQ1, “earlyborn”); April to June, second quartile (BQ2); July to September, third quartile (BQ3); and October to December, fourth quartile (BQ4, “lateborn”). This method of division has previously been validated in various athletes [5, 23] and soccer players [24]. The occurrence of the RAE was analyzed across all national team players, born in various quarterly periods, and across each age group (male: U-16 to senior; female: U-16 to senior).

### Measures

Two independent experts examined birthdate information of the national team squads. The data was commercially, and freely available, thus ethical approval and written consent was not required. However, the study adhered to the principles of the Declaration of Helsinki and received approval from the national association [25]. To maintain confidentiality, all data underwent anonymization prior to analysis.

### Statistical Analysis

Frequency counts were used to evaluate the distribution of players across birth quartiles (BQ1–BQ4) in male and female national squad players. Chi-square Goodness-of-Fit tests (χ^2^) and Cramer’s V effect size were employed to assess deviations from an expected uniform quartile distribution (25% per quartile). Cramer’s V thresholds were defined as: V ≤ 0.06 (trivial effect), 0.06 < V ≤ 0.17 (small effect), 0.17 < V ≤ 0.29 (medium effect), and V ≥ 0.29 (large effect). Odds ratios (ORs) and 95% confidence intervals (95% CIs) were calculated to compare BQ1&2 with BQ3&4, analyzing the likelihood of being born in the first versus the second quartile. Relative age effects were examined separately for male and female groups and across age categories (U-16, U-17, U-19, U-21, and senior teams). In addition, a chi-square test of independence was conducted to examine whether the distribution across quartiles (BQ1–BQ4) differed significantly between male and female players within each age group (U-16, U-17, U-19, and senior team). The evolution of RAEs in senior male and female groups was analyzed over a twenty-year period, starting from 1845. Youthto-senior transition rates were calculated to determine the proportion of players debuting in the senior team from each birth quartile, and according to the varying youth teams. These rates were assessed for male and female groups using binomial proportion confidence intervals, specifically applying the Clopper-Pearson method (90% CI).

To explore factors influencing the likelihood of transitioning to the senior team, a binary logistic regression was conducted. The dependent variable was dichotomous (0 = no transition; 1 = transition), with independent variables including (1) the number of youth team appearances (continuous), (2) birth quartile (BQ1–BQ4), and (3) player sex (1 = male; 0 = female). An interaction term between sex and youth team appearances was included to examine differences in the effects between male and female players. To examine the discriminatory power of the logistic regression model, the Area Under the Receiver Operating Characteristic Curve (AUC-ROC) was computed. An AUC of 0.5 suggests no discrimination, between 0.51 and 0.69 is considered poor discrimination, 0.70 to 0.79 is acceptable, 0.8 to 0.9 is considered excellent, and more than 0.9 outstanding [26]. All statistical analyzes were performed using Anaconda (Version 3.9.12, Anaconda Inc., New York, NY, USA) and relevant Python libraries.

## RESULTS

**FIG. 1:**
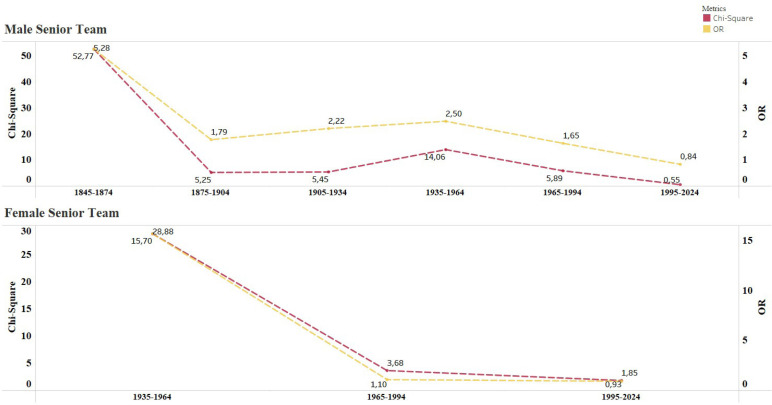
Evolution of the relative age effect (RAE) over a 30-year time period, assessed through chi-square (χ^2^) and odds ratio (OR) analyzes for male and female senior teams.

**FIG. 2:**
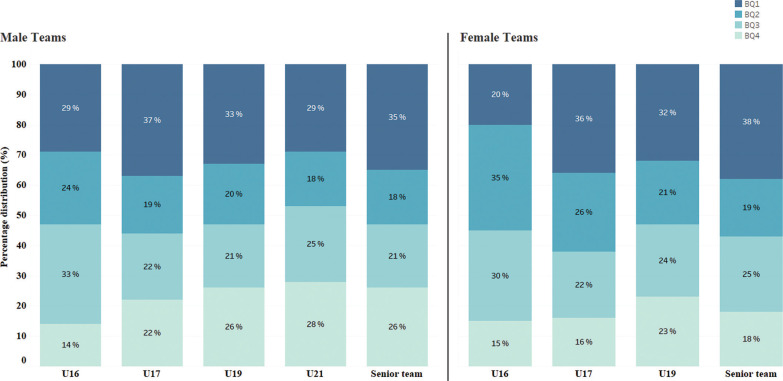
Distribution of players across birth quartiles (BQ) for male and female teams.

A total of 862 different male players were selected for national youth teams during the study period, whilst only 652 players participated in senior team matches. Although, 188 players transitioned from the youth teams. In the female category, 375 players participated in youth team matches, whilst 182 played at senior level, with 112 completing the transition from youth teams. Table 1 summarizes the RAE distribution, including chi-square analysis, Cramer’s V effect sizes, and odds ratios (ORs; BQ1&2 vs. BQ3&4) for male and female groups across U-16, U-17, U-19, U-21, and senior team categories. Significant RAEs were observed in all age categories except male U-16, female U-16, and female U-19. The effect sizes ranged from small to medium (Cramer’s V: 0.10–0.19). Odds Ratio analysis revealed a disproportionate representation of players born in BQ1&2 vs. BQ3&4, particularly evident in the senior teams. Male and female senior teams exhibited ORs of 2.27 and 1.50, respectively. Figure 1 demonstrates the evolution of the RAE, showing how chi-square values and ORs (BQ1&2 vs. BQ3&4) for male and female senior players. Figure 2 exhibits the birth quartile distributions according to the various youth teams. The chi-square analysis between male and female players within each age group showed no statistically significant differences.

**TABLE 1:** Relative age distribution, chi-square (χ^2^), odds ratio (OR), and distribution differences for male and female players across age groups.

Sex	Age-group	Total N.	BQ1 %	BQ2 %	BQ3 %	BQ4 %	χ2	p-value	Cramer’s V	OR BQ1&2 vs. BQ3&4	Male vs. Female (p–value)
Male	U-16	21	29	24	33	14	1.67	0.644	0.16	0.51 [0.08–3.11]	0.85
	U-17	501	37	19	22	22	35.33	< 0.001	0.15	1.87 [1.30–2.69]	0.08
	U-19	447	33	20	21	26	18.74	< 0.001	0.12	2.05 [1.40–2.99]	0.73
	U-21	415	29	18	25	28	12.73	0.005	0.10	1.82 [1.23–2.70]	–
	Senior team	652	34	18	21	26	37.51	< 0.001	0.14	2.27 [1.65–3.11]	0.14

Female	U-16	20	20	35	30	15	2.00	0.572	0.18	0.28 [0.04–1.82]	–
	U-17	239	36	26	22	16	20.63	< 0.001	0.17	0.99 [0.58–1.69]	–
	U-19	256	32	21	24	23	7.41	0.060	0.09	1.47 [0.89–2.41]	–
	Senior team	182	38	19	25	18	19.54	< 0.001	0.19	1.50 [0.82–2.77]	–

*The “Male vs. Female” column reports chi-square results comparing quartile distributions between sexes within each group. BQ = birth quartile; χ^2^ = chi-square; OR = odds ratio.

Table 2 presents the overall youth-to-senior transition rates by birth quartile for male and female players, whilst Table 3 highlights the transition rate according to the differing age group youth teams. The overall transition rate was relatively low, although higher in female players (30%) compared to male players (20%). However, male players reported the lowest transition rate in BQ1 (17%), whilst female players registered the lowest rate in BQ4 (25%). Furthermore, U-21 male (43%) and U-19 female (41%) players showed the highest transition rate across the age categories.

**TABLE 2:** Youth-to-senior transition rates for male and female players, presented overall and by birth quartile.

Sex	Birth Quartile	Total	Transition to the Senior Team	Transition rate % [CI 90%]
Male	BQ1	277	49	17 [14–21]
	BQ2	152	38	25 [19–31]
	BQ3	208	47	22 [18–27]
	BQ4	225	54	24 [19–29]
	**Overall**	**862**	**188**	**22**

Female	BQ1	121	37	30 [24–38]
	BQ2	89	28	31 [23–40]
	BQ3	86	27	31 [24–40]
	BQ4	27	20	25 [18–34]
	**Overall**	**375**	**112**	**30**

BQ = birth quartile; CI = confidence interval.

**TABLE 3:** Transition rates for male and female players, reported according to vaerying youth teams.

Sex	Youth Team	Total	Transition to the Senior Team	Transition rate % [CI 90%]
Male	U-16	21	0	0 [0–12]
	U-17	501	70	13 [11–17]
	U-19	446	81	18 [15–21]
	U-21	413	179	43 [39–47]

Female	U-16	20	1	5 [1–20]
	U-17	236	57	24 [19–29]
	U-19	256	105	41 [35–46]

CI = confidence interval.

Table 4 displays the results of the binary logistic regression analysis. The logistic regression showed an acceptable discrimination ability (AUC = 0.71). The number of youth team appearances was identified as a significant predictor of senior team transition probability. The β coefficient (0.177) indicates that each additional appearance increases the likelihood of transition to the senior team by 19.4%. Birth quartile and player sex did not have a significant effect. However, a significant interaction between sex and youth team appearances was identified. The negative β coefficient for the interaction term (-0.122) suggests that the impact of youth team appearances on senior team transition is less pronounced for male players compared to female players. Table S1 in the supplementary material summarizes the distribution of players by sex, age group, birth quartile, and transition from youth to the senior team.

**TABLE 4:** Results of binary logistic regression analyses.

Variables	β coefficient	CI 95%	Standard error	p-value
Constant	-2.626	-3.203 to -2.049	0.294	< 0.001
Presences in the Youth Teams	0.177	0.127 to 0.228	0.026	< 0.001
BQ2	0.347	-0.044 to 0.739	0.200	0.082
BQ3	0.362	-0.014 to 0.739	0.192	0.059
BQ4	0.363	-0.011 to 0.737	0.191	0.057
Sex	0.524	-0.056 to 1.105	0.296	0.076
Interaction (Sex × presences)	-0.122	-0.177 to -0.069	0.027	< 0.001

BQ = birth quartile; CI = confidence interval.

## DISCUSSION

The present study aimed to: (1) investigate the prevalence and magnitude of the Relative Age Effect (RAE) across a national team program; (2) assess the transition rates from youth to senior international level, and (3) examine the influence of birth quartile distribution on transition. The main findings revealed a significant RAE across most age groups, except for male U-16 and female U-16 and U-19 categories, where the effect size ranged from small-to-medium. Transition rates from youth to senior national team level was generally low, with female players exhibiting a higher transition rate (30%) compared to males (20%). However, contrasting trends were observed in birth quartile distribution, with the lowest transition rate occurring among BQ1 males (17%) and BQ4 females (20%).

Notably, the number of national youth team appearances emerged as a significant predictor of transition probability to the senior team, accounting for 19.4% of the variance (β = 0.177), with this relationship being more pronounced in female players than male players. These results highlight the complex interplay between relative age, sex, and developmental pathways in elite sport.

### Prevalence and Magnitude of the RAE

The present study findings demonstrate significant RAE across several age categories in national youth soccer teams. The observed effect sizes (Cramer’s V range: 0.10–0.19) indicate small-to-medium magnitude effects, consistent with previous reports from elite youth development systems [15, 27]. These results corroborate extensive literature documenting the systematic over-representation of athletes born earlier in the selection year [1–2]. Notably, non-significant RAE in male U-16 (*p* = 0.21), female U-16 (*p* = 0.34), and female U-19 (*p* = 0.12) cohorts were found, suggesting important developmental variations across age groups and sex.

The manifestation of RAE in the current sample aligns with existing international patterns observed in elite youth soccer. Comparative data from FIFA tournaments reveal consistent over-representation of early-born players, with 40.6% of U-17/U-20 World Cup participants (2009–2019) born in BQ1 versus only 16.9% born in BQ4 [28]. Historical analysis shows similar distribution patterns in U-17 World Cups [20], whilst recent longitudinal data confirm these trends persist across U-17 to U-21 competitions [29]. These consistent findings across studies and competitions underscore the robustness of the RAE phenomenon in elite youth soccer.

The absence of significant RAE in U-16 teams of both sexes may reflect developmental selection practices that prioritize technical potential over physical attributes during early adolescence. This observation aligns with research demonstrating that youth selectors may show greater tolerance for physical immaturity when assessing technical proficiency [30]. Such selection approaches could help mitigate the RAE during critical early developmental stages when physical differences are most pronounced.

At the U-16 level, international soccer is framed by governing bodies and the international community as a platform for development rather than competitive outcomes. For instance, UEFA designates these tournaments as International Development Tournaments, deliberately de-emphasizing winning in favour of player development. This approach may grant coaches greater flexibility in squad selection, prioritizing long-term senior international potential over current performance. Such a shift could help mitigate selection bias toward more physically advanced players and reduce the RAE. The absence of a pronounced RAE in male and female U-16, and female U-19 players may reflect this developmental tolerance, early dropout of less physically mature players, or sex-specific maturation patterns (with females generally maturing earlier and showing less variability in pubertal timing). Similar exceptions have been observed in comparative studies of youth sport systems that prioritize holistic development over immediate results [1, 4, 8, 15].

Furthermore, in 2024 UEFA introduced the Fitness Competency Framework and associated diploma [31], which includes modules on growth and maturation, highlighting the increasing recognition of these factors in player development. This proactive educational initiative provides coaches with a deeper understanding of how growth and maturation influences player progression.

For the examined female U-19 team, the null RAE contrasts with persistent effects in male cohorts and likely reflects earlier biological maturation in females, who typically complete peak height velocity around 11.5–12.0 years compared to 13.5–14.0 years in males [32]. By late adolescence, this reduces variability in physical development and may shift selection criteria toward technical and tactical qualities rather than physical dominance. This aligns with evidence of diminished RAE effects in collegiate women’s soccer [33, 34].

The senior national teams exhibited the most pronounced RAE (male OR = 2.27, female OR = 1.50), suggesting that cumulative advantages persist into elite adulthood, and recent developmental interventions may not yet significantly influence senior team selection. These findings align with a cascading advantage framework [35] wherein initial relative age advantages lead to enhanced selection probability during critical periods (ages 6–10 years), greater access to developmental resources during pre-adolescence (ages 11–14 years), and sustained psychological and performance benefits through adolescence. The persistence of these effects at the senior level underscores the need for systemic interventions throughout athlete development pathways to mitigate RAE-related inequities.

### Evolution of the RAE over time

The analysis of the RAE over time, as depicted in Figure 1, reveals notable trends in both male and female senior teams. For male senior teams, the chi-square values and ORs have shown a consistent increase over a 30-year period, particularly from 1965–1994 and 1995–2024. This suggests that the RAE has become more pronounced in recent decades, potentially due to increased competitiveness, professionalization, and early specialization in sports [1–2]. Similarly, female senior teams exhibit a rising trend in the RAE, albeit starting from a lower baseline during the 1935–1964 period. The increasing ORs over time indicates that relatively older female players are increasingly over-represented in the senior teams, mirroring trends observed in male soccer cohorts.

These findings collaborate previous research [15] highlighting the persistence and escalation of the RAE in elite sports. Its evolution may reflect systemic biases in talent identification processes, particularly as modern youth academies commence scouting as early as 6–10 years old. The resulting concentration of coaching resources and opportunities for selected players may perpetuate this bias longterm. Notably, the stabilization of chi-square values in recent years for female national teams (1995–2024) could indicate a growing awareness of the RAE and efforts to mitigate its impact, though further research is required to confirm this hypothesis.

### Youth-to-Senior National Teams Transition Rates

The transition rates from youth to senior national teams, as shown in Tables 2 and 3, provide critical insights into the existing pathways for talent development. Overall, there was a marked disparity in transition rates between female (30%) and male (20%) players, which may reflect differences in competition depth, developmental trajectories, or selection criteria between sexes [35]. Scandinavian models demonstrate significantly later competitive stratification in women’s soccer, with selection pressures emerging at U-17 compared to U-12 in male pathways [11], whilst English FA data reports females’ academies allocate 23% more training time to technical versus athletic development [34], which may reduce reliance on physical maturation advantages. These structural differences may help explain the contrasting birth quartile patterns. Male BQ1 lower transition rates (17%) mirror European trends where early-selected males demonstrate 21–28% higher dropout rates by U-19 [27] likely due to premature specialization prior to peer catch-up, sited as the ‘big-fish-little-pond’ effect [36], where early selection advantages may inadvertently limit long-term development through reduced competitive challenges. Conversely, female BQ4 players’ maintained transition rate success (25%) which corresponds with Swedish studies showing late-born athletes developing compensatory technical advantages by senior level [37]. These findings suggest that female development systems mitigate the RAE through later selection timing and technically focused training, thus creating more balanced pathways for earlier biological selection. Moreover, the relatively higher female BQ4 transition rate overall points to the effectiveness of women’s development structures in retaining and advancing talent, with important implications for sustaining growth and competitiveness in the female game.

Among the national youth teams examined, the highest transition rates were observed in male U-21 (43%) and female U-19 (41%) players. These findings highlight the importance of late-adolescent and early adult development stages in talent identification and retention. The higher transition rates in these age groups may reflect the maturation of technical, tactical, and physical attributes required for senior-level competition [38]. However, the low transition rates in younger age groups (e.g., male U-16 and female U-16) highlights the challenges of early talent identification and the need for longterm development strategies.

### Predictors of Transition to Senior Teams

The binary logistic regression analysis (Table 4) identified the number of youth team appearances as a significant predictor of transition rate to the senior team probability, with each additional youth appearance increasing the likelihood of progressing to the senior team by 19.4% (β = 0.177, *p* < 0.01). This finding emphasizes the importance of consistent playing opportunities in fostering player development and transition to an elite senior level [39].

Recent research examining Japanese soccer academies highlighted similar findings [40], underscoring the importance of embedding club philosophies that prioritize long-term player development over short-term results. This aligns with the Football Association of Wales’s recent emphasis on creating a robust games program across all youth age groups, that ensures both a high volume of competitive matches and exposure to quality opponents. By providing promising young players with international experience and mirroring the senior national team’s playing model, the program facilitates a smoother transition to elite-level competition. Crucially, the focus remains on offering developmentally appropriate opportunities rather than fast-tracking players who may not be ready to contribute at the senior level. Notably, birth quartile and player sex were not significant predictors of senior transition. This suggests that while the RAE may influence early selection into youth squads, it does not determine long-term success or the probability of reaching senior international level.

A significant interaction between sex and youth team appearances (β = -0.122, *p* = 0.03) indicates that the impact of playing opportunities is less pronounced for male players compared to females. The attenuated effect of appearances for males may reflect a ceiling effect. For example, in the present study, male players averaged 18.7 caps versus females’ 12.9, although the transition rate was lower (17% vs. 30%). These findings mirror English Premier League findings where more than 15 accumulated international appearances yielded diminishing returns (r^2^ = 0.11 [41]), whereas in the female game, each additional appearance increased senior debut odds by 27% (OR = 1.27, 95% CI:1.09–1.48). This may reflect differences in competition structures, with male players facing greater depth and variability in talent pools, thereby reducing the relative advantage of additional appearances. For female players, consistent playing time appears to be a more critical factor in facilitating transition, potentially due to smaller talent pools or fewer competitive opportunities at the senior level.

### Practical Implications and Recommendations

The findings of this study have several practical implications for talent identification and development programs. Firstly, the persistent RAE across age groups and its evolution over time highlight the need for systemic interventions to ensure equitable opportunities for all athletes, regardless of sex and birthdate. Strategies such as rotating selection dates, implementing bio-banding, or focusing on skill development rather than short-term performance and match outcomes may help mitigate the RAE [42]. However, the feasibility of these interventions varies by national context and may not be fully applicable within the examined national structure. National associations could also explore quota systems, flexible age-banding, or biologically informed developmental benchmarks to counteract systemic bias. Secondly, the higher transition rates in late-adolescent and early adult age groups (e.g., male U-21 and female U-19 teams) suggests that national talent identification programs should prioritize long-term development over early selection to provide consistent playing opportunities, particularly for female players, and enhance transition rates and reduce dropout rates among talented athletes. Finally, the interaction between sex and youth team appearances highlights the need for sex-specific approaches to talent development. Whilst increased playing time is crucial for both male and female players, the differing impact of the RAE, which was significant in all age groups except the U-16 male and U-16 and U-19 female groups, suggests that tailored strategies may be necessary to optimize transition rates for each both male and female groups.

### Limitations and Future Research Directions

Several limitations must be acknowledged when interpretating the findings of this study. Firstly, the focus of only a single national association may limit the generalizability of the findings to other contexts or sports. Secondly, data should be interpreted considering the temporal distribution shown in Figure 1. The observed imbalance arising from factors such as incomplete records in the most recent years and historical sex disparities at the onset of data collection must be considered when drawing conclusions and attempting to practically apply such findings. These discrepancies may influence the representativeness of the sample across decades and between sexes, potentially affecting the robustness of temporal or sub-group comparisons. Thirdly, only birth dates were recorded, omitting essential anthropometric measures, maturation status, and performance variables, such as physical and technical/tactical match performance data, and fixture outcome, crucial for a comprehensive understanding of physical status and the RAE [4]. Finally, the study did not account for potentially confounding variables such as injury rates, coaching quality, or socio-economic factors, which may influence player development and transition. Future research should explore the impact of the RAE in different cultural contexts (other national teams from a variety of regions across the world) and other sports, investigate the role of psychological and social factors in player transitions, and evaluate the effectiveness of interventions to reduce the RAE. Longitudinal studies tracking player development from youth to senior level may also provide further insights into the mechanisms underlying the RAE and its long-term consequences.

## CONCLUSIONS

In conclusion, this study highlights the pervasive nature of the RAE in a national team structure and its impact on transition rates to the senior team. While the RAE is observed across most age groups, its magnitude and implications vary by sex and developmental stage. Consistent playing opportunities emerged as a critical factor in facilitating transition to senior teams, particularly for female players. Addressing the RAE and optimizing talent development pathways will require systemic changes, sex-specific strategies, and a focus on long-term player development.

## Supplementary Material

The relative age effect and transition rates across a national soccer program in male and female youth to senior players. A longitudinal analysis
