# Supplementary material for: The relative age effect and transition rates across a national soccer program in male and female youth to senior players. A longitudinal analysis
Source: Biol Sport. 2025 Oct 1;43:439–48. doi: 10.5114/biolsport.2026.154941 (PMC12954492; doi:10.5114/biolsport.2026.154941)
Supplement: The relative age effect and transition rates across a national soccer program in male and female youth to senior players. A longitudinal analysis [file JBS-43-56929-s1.pdf]

S1 TABLE. Distribution of players by sex, age group, birth quartile (BQ), and transition to the first team. The number of appearances for each group is also reported.

Sex	Age-group	Birth Quartile	Transition to the first team	N. of players	N. of appearances
Male	U-16	BQ1	Yes	0	0
			No	6	50
		BQ2	Yes	0	0
			No	5	44
		BQ3	Yes	0	0
			No	7	48
		BQ4	Yes	0	0
			No	3	12
	U-17	BQ1	Yes	24	614
			No	158	1650
		BQ2	Yes	18	410
			No	79	940
		BQ3	Yes	12	206
			No	99	951
		BQ4	Yes	16	354
			No	95	894
	U-19	BQ1	Yes	26	650
			No	121	1494
		BQ2	Yes	21	510
			No	68	887
		BQ3	Yes	13	216
			No	80	867
		BQ4	Yes	21	428
			No	96	988
	U-21	BQ1	Yes	47	754
			No	71	858
		BQ2	Yes	35	536
			No	38	613
		BQ3	Yes	45	433
			No	58	594
		BQ4	Yes	52	618
			No	66	665
Female	U-16	BQ1	Yes	1	11
			No	3	21
		BQ2	Yes	0	0
			No	7	44
		BQ3	Yes	0	0
			No	6	28
		BQ4	Yes	0	0
			No	3	19
	U-17	BQ1	Yes	20	300
			No	66	489
		BQ2	Yes	15	179
			No	47	335
		BQ3	Yes	15	175
			No	36	295
		BQ4	Yes	8	84
			No	29	239
	U-19	BQ1	Yes	36	450
			No	46	398
		BQ2	Yes	25	266
			No	28	233
		BQ3	Yes	26	274
			No	36	308
		BQ4	Yes	18	166
			No	41	289

BQ = birth quartile.

Articles published in the Biology of Sport are licensed under an open access Creative Commons CC BY 4.0 license.
